# Plants and endophytes – a partnership for the coumarin production through the microbial systems

**DOI:** 10.1080/21501203.2022.2027537

**Published:** 2022-02-07

**Authors:** Chandrashekar Srinivasa, Govindappa Mellappa, Shashank M. Patil, Ramith Ramu, Bhargav Shreevatsa, Chandan Dharmashekar, Shiva Prasad Kollur, Asad Syed, Chandan Shivamallu

**Affiliations:** a Department of Studies in Biotechnology, Davangere University, Davangere, India; b Department of Studies in Botany, Davangere University, Davangere, India; c Department of Biotechnology and Bioinformatics, School of Life Sciences, JSS Academy of Higher Education and Research, Mysuru, India; d Department of Sciences, Amrita School of Arts and Sciences, Amrita Vishwa Vidyapeetham, Mysuru Campus, Mysuru, India; e School of Agriculture, Geography, Environment, Ocean and Natural Sciences (SAGEONS), The University of South Pacific, Suva, Fiji; f Department of Botany and Microbiology, College of Science, King Saud University, Riyadh, Saudi Arabia

**Keywords:** Endophytic fungi, coumarin, host–endophyte interaction, secondary metabolites

## Abstract

Plant-based secondary metabolite production system is well established. However, host–endophyte interaction in the production of secondary metabolite is a new less exploited area that is overcoming barriers and evolving as one of the prospective fields. Endophytes such as bacteria or fungi have the ability to produce some of the secondary metabolites that mimic the plant metabolites therefore escaping the host defence system. Coumarin is one such metabolite with immense biological functions. Most of the studies have demonstrated coumarin production from fungal endophytes but not bacterial endophytes. Herein, we present an overview of all the coumarin derivatives produced from endophytic sources and their biosynthetic pathways. Furthermore, the review also throws light on the isolation of these coumarins and different derivatives with respect to their biological activity. The biotransformation of coumarin derivatives by the action of endophytic fungi is also elaborated. The present review provides an insight on the challenges faced in the coumarin production through fungal endophytes.

## Introduction

An intricate cost–benefit plant-microbe association that remains symptomless and unobtrusive inside the plant tissues is represented as endophytism. This phenomenon is described as the ability of the microorganism to remain within the plant host without discernibly harming the host (Salvatore et al. 2020). An amalgamation of an extremely committed mutualism and ardent parasitism and saprophytism causes specialised interactions between the microbe and its host plant. Studies have proven the association of plants with microbes right from the first appearance of plants on earth. Studies by Guerin (1898) identified the first fungi associated with plant; de Bary (1866) coined the term endophyte referring to the microorganisms present in the plant tissues. The discovery of the presence of *Neotyphodium coenophialum* in the grass *Festuca arundinacea* is marked as a milestone in the endophyte research that was identified to cause fescue toxicosis in cattle (Bacon et al. 1977). Eventual research identified that in fact these plants contained toxic alkaloids that were the cause of the disease and that the fungi itself was imparting tolerance to the plant from biotic and abiotic stress (Schardl et al. 2004).

Similar studies towards understanding this multispecies crosstalk have led to direct effects on the organisms’ phenotype and functional trait. As a result of this crosstalk, a large amount of complex metabolic flux occur leading to the production of several primary and secondary metabolites that contain a huge medicinal and pharmacological significance (Borges et al. 2009; Debbab et al. 2013; Mousa and Raizada 2013). A large number of bioactive substances are produced by the endophytic microorganisms with potential therapeutic applications. Some of the popular metabolites produced from these endophytes include azadirachtin (Shirahatti et al. 2015), paclitaxel or taxol (Stierle et al. 1993), podophyllotoxin, hypericin, and emodin (Srikantaramas et al. 2007). The production of secondary metabolites is not a mere biochemical reaction but impacted at the molecular level and thus has a significant impact from the ecological standpoint. A plethora of novel bioactive compounds have been isolated and characterised from these endophytes and therefore attract enormous research on these lines. Variations in parameters such as pH, temperature, agitation, media composition, pO_2_, pCO_2_, and other physiological components result in the optimisation of the metabolite production (Stierle et al. 1993). Yet, the feasibility of their commercial production is still under question and requires attention. One of the key challenges that need to be addressed is the reduction of metabolite production owing to continuous subculturing of the axenic monoculture (Salvatore et al. 2020). To add to the woes, discoveries of the presence of secondary metabolites from such endophytes have mostly been performed from monocultures, and thus, the current understanding is insufficient to optimise the same for large-scale production (Guerin 1898).

In this regard, coumarin, also known as 2 H-chromen-2-one, is an aromatic organic molecule having the formula C9H6O2. Its structure is a benzene molecule with two neighbouring hydrogen atoms substituted by a lactone-like chain − = −−O−, resulting in a second six-membered heterocycle sharing two carbons with the benzene ring. Coumarins and its derivatives are biosynthetically, structurally, and pharmacologically interesting natural compounds (C1–C17). They’ve been found in plants, microorganisms, marine organisms, bacteria, insects, liverworts, and fungi, among other natural sources (e.g. soil, endophytic, and marine fungi). They are the key intermediates in the synthesis of several carbo- and heterocyclic compounds, including isoquinolines, isochromenes, and many aromatic compounds. As a result, the coumarin framework has been investigated in a variety of fields, including drug development, pharmaceutical and medicinal chemistry, and chemical synthesis. Antimicrobial, cytotoxic, algicidal, antiallergic, immunomodulatory, antimalarial, plant growth regulating, and acetylcholinesterase and protease inhibitors are among the bioactivities documented for these compounds. Considering their various potential for its pharmacological and bioactive properties, in the present review, we emphasise on the plant–endophyte interaction as a key for the production of secondary metabolites, the challenges faced in the large-scale production as well as perspectives to overcome these challenges with special emphasis on the production of coumarin as a secondary metabolite by the endophytes.

### Plant and endophytes are equal partners for secondary metabolite production?

**Figure 1.**
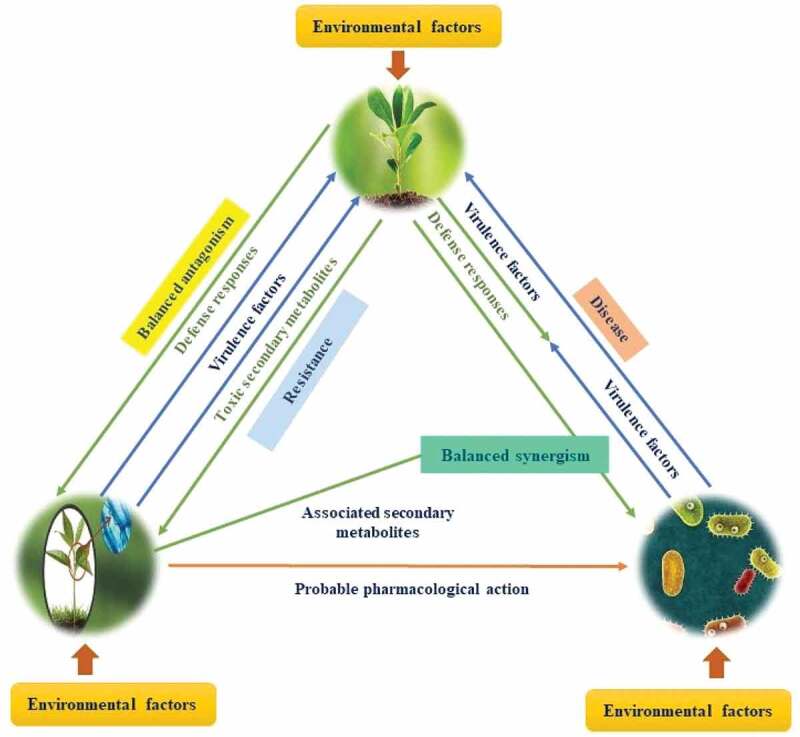
Mechanism of balance seen in the interaction between the plant and endophyte.

Once a plant encounters an endophyte, be it a bacterium or a fungus, it precedes several physiological reactions as a result of its interaction with the physical and chemical barriers within the plant system. One hypothesis that states the mechanism employed by the endophyte to deviate from the host-immune response is known as the balanced antagonism theory. This theory is indicative that the endophyte extends self-resistance upon its first contact with the host before being incapacitated by the secondary metabolites of the host system (Kusari et al.). It devices a careful balance between the defence factors present within both the endophyte as well as the plant system. This balance between the two remains asymptomatic but is likely to be a transitory phase that can be escalated to virulent, symptomatic condition if triggered by several environmental factors. These factors play a pivotal role in maintaining this balance. However, minute trigger in the environmental factors can create an imbalance causing the avirulent interaction to turn into a pathogenic one, thus eliciting the host defence cascade. If the plant is capable of fighting the pathogen, it leads to the elimination of this endophyte-turned pathogen. But, if it is unable to fight the pathogen it succumbs to the virulence leading to plant diseases Kusari et al.). Figure 1 represents the mechanism of balance seen in the interaction between the plant and endophyte. For instance, expression of sakA, a mitogen-activated protein kinase gene, in the endophytic fungi *Epichloe festucae* in required for its interaction with the host *Lolium perenne*. This expression converts the endophytic interaction into a pathogenic one (Debbab et al. 2013).

However, studies reveal more complex phenomena controlling this interaction with intricate details. Recent study carried out by Srikantaramas et al. (2007) revealed that a plant product camptothecin produced by *Camptotheca acuminata* is also produced by its endophyte *Fusarium solani*. The role of this plant metabolite is known to inhibit topoisomerase I by associating with the complex formed between DNA and the enzyme. However, camptothecin produced by the endophyte imparts protection from the plant metabolite by altering specific amino acid residue on its topoisomerase I. A similar alteration was also observed in the topoisomerase I obtained from another endophyte thriving within the same plant, suggesting this modification of the endophyte to be against the host secondary metabolite (Guerin 1898). This demonstrates that while the plant employs secondary metabolite production as a mechanism to counter predation, the endophyte has evolutionarily adapted itself to prevent from being eliminated from the host. Without this intrinsic ability, the invading fungus is most likely to be killed by the action of camptothecin. Herein, the fungi should be programmed to prevent the attack by camptothecin before biosynthesis of its own. A similar camptothecin resistance is also observed in certain plants such as *Ophiorrhiza japonica* that possesses partial resistance against camptothecin inhibition by the modification of a few amino acids. However, this plant does not itself produce camptothecin, suggesting complex unknown mechanisms contributing to the imparted resistance (Srikantaramas et al. 2007). The concept of co-evolutionary adaptation does not explain the driving force for such an adaptation in this plant. However, such adaptations are observed only in case of plants probably due to the presence of co-evolution that ensure the survival of endophytic organisms within the plant species.

Conversely, some of the theories indicate that the presence of endophyte drives the plant towards producing such metabolites as chemical responses to acknowledge its presence although it cannot be classified under host defence response. In this regard, two parallel theories have been proposed. The ‘mosaic effect’ suggests that the plant is in fact being protected by the endophyte by making a genetically homogeneous chemical composition within different organs of the plant into a heterogeneous mixture upon expression (Borges et al. 2009). Conversely, the xenohormesis theory states that the endophytes are capable of sensing the production of stress-induced chemicals and possesses memory for the production of similar chemicals and metabolites to prevent its own elimination from the host (Kusari et al.). It is due to this reason the gene clusters that are employed in the biosynthesis of such metabolites have remained homologous throughout the evolutionary development.

### Endophytes are a treasure of bioactive products

Chemical metabolites are constantly produced by the interaction of the plant with its endophyte. As stated in the previous section, although the exact driving force for the production of these chemicals are not clearly understood, it can be stated that both the plant as well as the endophyte takes the onus of producing these metabolites as a response to any change in the environmental conditions. Research has facilitated the production of these chemicals at an industrial scale by isolation of such endophytes and presenting optimal environment for its biosynthesis. Bioprocessing of fungal endophytes produces enormous physiologically active compounds such as antibiotics, antibodies, plant hormones, growth promoters, statins, terpenes, alkaloids, phenolics, steroids, drugs with anticancer, antitumor, antioxidant, hypercholesterolemia, antidiabetic, and several other bioactives (Hansen et al. 2015). Among the various chemical entities that are produced by these endophytes, secondary metabolites are of greater significance as the organisms can be manipulated for their production at an industrial scale.

Isolation of endophytes from the host tissues has been a successful strategy to first obtain a pure culture of the endophyte, which is further processed to produce the desired metabolite by exposing them to optimal conditions (Valdez-Nuñez et al. 2019). Increasing number of research is shifting from plant-based products to the isolation of bioactive principles from endophytes. Over the years, despite a huge attention in this field only about 0.75–1.5% of the plant–endophytic interactions have been identified among over 400,000 plant species known (Stierle et al. 1993; Borges et al. 2009). The first of its kind research in the identification of chemical resources from endophytes was the discovery of taxol by Stierle et al. (1993) from the endophytic fungi *T. andreanae* way back in 1993. This was followed by a series of studies to identify a large number of compounds that were thought to be produced by the plant hosts but were in fact being produced by the endophyte thriving within the host system. Therefore, such metabolites can be classified under plant/host-derived metabolites that are recently established as co-produced metabolites. Owing to the fact that medicinal plants are an inherent source of such bioactive compounds, it becomes highly important to assess these plants and their endophytes to obtain them at an industrial scale.

### Overview of plant-driven bioactives from endophytes

**Figure 2.**
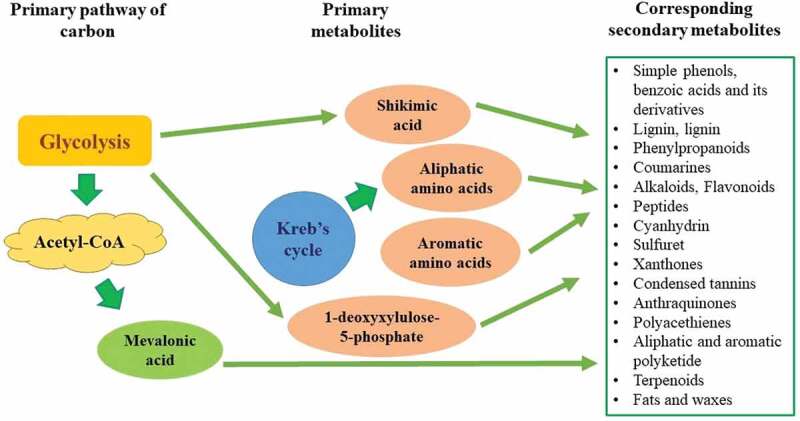
An overview of all the secondary metabolites and corresponding pathways for its production.

Secondary metabolite production is a routinely active pathway within all living systems that work in response to any changes in the external conditions. Endophytic microbes also produce a wide array of such metabolites and can be classified as alkaloids, terpenes, non-ribosomal peptides, polyketides, and a few others. Alkaloid biosynthesis occurs through the shikimic acid and mevalonate pathways from the aromatic amino acids and dimethylallyl pyrophosphates, whereas terpenes containing isoprene units are synthesised through the mevalonate pathway by the action of the enzyme terpene cyclase. Similarly, non-ribosomal peptide biosynthesis takes place via both proteinogenic and nonproteinogenic amino acids and polyketides are produced by the respective synthases from the precursor acetyl coA and malonylcoA (Stierle et al. 1993). With these constituting major pathways of secondary metabolite production, the other chemicals are produced by linking to one of these pathways. Overall, the secondary metabolites are classified as alkaloids, coumarins, flavonoids, lignans, saponins, terpenes, quinones, and xanthones, and miscellaneous compounds. The figure represents an overview of all the secondary metabolites and corresponding pathways for its production (Figure 2).

### Coumarins- a scaffold with enormous biological activity

Among the various bioactive metabolites produced from endophytic microorganisms, coumarins constitute an important class owing to their versatile biological properties. This class of compounds has been identified from plant and microbial origin. They are the secondary metabolites with a wide array of biological activity used in food and cosmetic industries. Coumarins are the aromatic compounds produced through the shikimic acid pathway via phenylalanine metabolism. The simplest member of the coumarin family was first a plant derived compound obtained from *Coumarouna odorata* isolated by Vogel in 1820 (Guerin 1898). They are chemically made of a benzo-α-pyrone (2 H-1-benzopiran-2-one) nucleus. They have been classified as simple coumarins, furanocoumarins, pyranocoumarins, biscoumarins, triscoumarins, and coumarinolignans. They are biologically important molecules owing to the characteristics such as high solubility, simple structure, low molecular weight, and low toxicity, thus facilitating them to possess potent pharmacological properties. They are also used as additives in the food industry owing to their characteristic aroma. Some of the derivatives of this class of compounds possess potent luminescent properties.

### Coumarins from endophytes

The first isolation of coumarins was from the seeds of *Coumarouna odorata*, which derived the nomenclature of this class of compounds as coumarins (Srikantaramas et al. 2007). Fungal endophytes have been a rich source of various secondary metabolites. They produce them as a response to their interactions with the host plant and are specific to a particular host and environmental conditions. Overall, seven such coumarins have been identified from the fungal sources to date that are summarised in Table 1.

**Table 1.** Details of various coumarin derivatives isolated by fungi and their host plant with special emphasis on their biological activity.

Coumarin derivative	Endophyte source	Host plant	Biological activity	Reference
Isofraxidin	*Alternaria alternata, Arcopiluscupreus, Aspergillus niger, and Corynespora cassiicola*	*Schleicheraoleosa* tissues	Antibacterial activity against *Escherichia coli, Klebsiella pneumoniae, Pseudomonas aeruginosa, Pseudomonas syringae, Salmonella enterica, Salmonella typhi, Enterococcus faecalis, Staphylococcus aureus, and Xanthomonas campestris* Antioxidant activity using cyclic voltammetry method. Isofraxidin significantly inhibited hepatoma cell invasion, expression of 12-O-tetradecanoylphorbol-13-acetate (TPA)-induced matrix metalloproteinase-7 (MMP-7) therefore recognised as anticancer agent	Gagana et al. 2020 Yamazaki and Tokiwa 2010
p-coumaric acid	*Alternaria alternata*	*Catharanthus roseus* leaves	Antimicrobial (against an array of bacteria and fungi) and antimycotoxigenic (as anti-aflatoxin B1 and anti-fumonisin B1 agent) activity. Act as potent protective agents for preventing microbial deterioration and mycotoxins accumulation in food	Sudarshana et al. 2019
(Z)-N-(4-hydroxystyryl) formamide- a coumarin analogue	*Aspergillus fumigatus*	*Meynalaxiflora* tissues	Antioxidant and drought resistant properties. In vitro using DPPH and in vivo using SH-SY5Y cells.Drought resistance was more potent that of the standard osmotic modulator proline.	Qin et al. 2019
Armillarisin A	*Armillaria tabescens*	*Malus*sp	Regulate liver function by modulating bile production and secretion and reduce inflammation	Wang and Guo 2007
Coumarin	*Ganodermalucidum*	Unknown	Antibacterial activity against *Bacillus subtilis*	Islam et al. 2015
Pestalasin A, Pestalasin B, Pestalasin C	*Pestalotiopsis*sp., *Penicillium purpurogenum*	*Rhizophora mucronata*	Antimicrobial activity against *Bacillus subtilis, Staphylococcus aureus, Streptomyces viridochromogenes, Escherichia coli, Candida albicans, Mucormiehei, Chlorella vulgaris, Chlorella sorokiniana, Scenedesmus subspicatus, Rhizoctoniasolani, Pythium ultimum*, and *Aphanomyces cochlioides*. *Cytotoxicity was evaluated against*	Xu et al. 2009; Shaaban et al. 2016
5,7-dimethoxy-4-p-methoxylphenylcoumarin or 5,7-dimethoxy-4-phenylcoumarin	*Streptomyces aureofaciens*	Root tissue of *Zingiber* *officinale* Rosc.	Showed a decrease in bcl-2 and an increase in Bax in A427 cell suggesting antitumor activity. 5,7-dimethoxy-4-phenylcoumarin showed better anti-tumour activity over that of 5,7-dimethoxy-4-p-methoxylphenylcoumarin	Taechowisan et al. 2007
Coumarin and ortho-coumaric acid	*A. niger, Fusarium oxysporum*	Different parts of *Crotalaria pallida*	Ortho-coumaric acid showed inhibition against the viral glycohydrolase enzyme (á-glucosidase, β-glucuronidase and lysozyme) *in vitro* suggesting anti-HIV activity.	Umashankar et al. 2012
Stem: furocoumarin, bark: coumarin (2 H-1-benzopyran-2-one), coumaric acid (3-benzofurancarboxylic acid), hymecromone (coumarin 4), 4-hydroxy-9-(3-methyl-2-butyl)furo(3,2-g)chloronen-7-one	*Fusarium equiseti,Cladosporiumuredinicola* and *Alternaria alternata*	Calophyllum tomentosum	Not reported	Melappa 2020
4-Hydroxycoumarin	*Niger*	Unknown host	Cytotoxic and spasmolytic activities, and also activity against HIV virus, and analgesic, anti-arthritis, anti-inflammatory, antipyretic, antibacterial, antiviral, and anti-cancer properties	Parshikov et al. 2015
(3 R*,4S*)-6,8-dihydroxy-3,4,7-trimethylisocoumarin and (3 R,4S)-6-8-dihydroxy-3,4,5-trimethylisocoumarin	*Penicillium sp.*	*Bruguiera sexangula*	Exerted moderate cytotoxic activity against tumour cell line K562	Han et al. 2009
Umbelliferone	*Annulohypoxylon ilanense*	*Taxuswallichiana* Zucc.,	Growth-inhibitory effects on human cancer cell lines including leukaemia	Nicoletti and Fiorentino 2015
7-Amino-4-methylcoumarin	*Xylaria sp. YX-28*	*Ginkgo biloba L.*	Antibacterial and antifungal activities *in vitro* against *Aeromonas hydrophila, Escherichia coli, Salmonella enteritidis, Salmonella typhi, Salmonella typhimurium, Shigella sp., Staphylococcus aureus, Yersinia sp., Vibrio anguillarum, Vibrio parahaemolyticus, Candida albicans, Aspergillus niger*, and *Penicillium expansum*. Anti-tubercularian activity against *M. tuberculosis*	Liu et al. 2008 Tandon et al. 2011

### Isolation and characterisation of coumarins

An ideal workflow for the production of bioactive compounds from endophytes can be collectively known as biotransformation or microbial transformation (Tian et al. 2021). The protocol includes the identification of endophytes producing bioactive compounds followed by its isolation. Isolation ideally employs surface sterilisation of the host parts such as twigs, leaves, stem, and seeds followed by placing them on enriched media. As coumarins and its derivatives have been isolated from various parts of the plants, their isolation also entails the collection of samples from different plant parts. This method is continued by mass cultivation of the potential microbe for product formation. Furthermore, various methods of extraction including different solvent system will result in the isolation of a series of chemical compounds (Schardl et al. 2004). Based on the bioactivity of the extract, the most potent extract can be obtained for further screening of individual chemical agent based on methods of characterisation such as UV-vis spectrophotometer, NMR, GS-MS, LC-MS, MS-MS, FTIR, XRD, and many other methods (Stierle et al. 1993). After characterisation, the isolated endophyte can be subjected to the process of fermentation for mass-cultivation of the desired product. This is conjugated with genetic manipulation methods for enhancing the product formation at some instances (Debbab et al. 2013). Overall, these steps constitute a general overview of the large-scale production of bioactive molecules from endophytic origin.

However, this workflow has several hurdles posed by certain factors such as seasonal dependency for the production of specific metabolites, ecological factors, and habitat of the host plant (Debbab et al. 2013). To improve yield of the metabolite desired, several methods with biotechnological applications are used as cost-effective and inexhaustible resource with high throughput bioactive compounds. Biotransformation methods are highly acclaimed for their applications in the production of antimicrobials (essential oils, vanillin), volatile compounds, antioxidants (eugenol, vanillin), anti-cancer agents (taxol), and anti-inflammatory agents (1,8-cineole) (Guerin 1898; Stierle et al. 1993; Debbab et al. 2013). Biotransformation employs the modification of molecules producing better functional groups with enhanced activity and bioavailability (Borges 2009).

Overall, the method for isolation and characterisation remains universal with minor modifications. The first criteria for optimal extraction are the use of appropriate media to culture the endophytic microbe. The cultures are ideally grown on potato dextrose agar, malt extract agar, or rice media that optimally supplements all the resources for the growth of the endophyte (Guerin 1898; Stierle et al. 1993; Srikantaramas et al. 2007). One of the most accepted solvents for the extraction of coumarins is ethyl acetate followed by methanol that has enabled optimal yield when compared to all the solvents (Stierle et al. 1993; Srikantaramas et al. 2007; Brader et al. 2014) demonstrated a microwave-assisted extraction method of coumarins that employed mixing of the fungal mat of *Alternaria* sp. with ethanol along with exposure to microwaves. This method relatively enhanced the yield of extraction of the secondary metabolite in the study. Several other studies also employ microwave-assisted extraction for better yield (Stierle et al. 1993; Debbab et al. 2013). The extracts are then subjected to spectrophotometric and other advanced biochemical assays for characterisation of the bioactive compound.

### Pharmacological potential of coumarins

#### Isofraxidin

Isofraxidin isolated from *Acanthopanax senticosus* (Siberian ginseng) and *Apium graveolens* is a hydroxycoumarin with important pharmacological potential. *Biscognia uxiacylindrospora* was the first fungal endophyte from which this metabolite was isolated. They exert their biological role on lipid metabolism by reducing the accumulation of triglycerides, release of TNF-α, enhancing the AMPK phosphorylation, activation of reactive oxygen species, and regulation of acetyl CoA carboxylase enzyme activity (Stierle et al. 1993). They also play a key effect on the expression of fatty acid synthase in liver and 3-hydroxyl-3-methylglutaryl-CoA synthase 2, thereby inhibiting lipogenesis. Overall, it controls the levels of lipids in circulation. They are also known as anti-inflammatory agents owing to their ability to downregulate TLRs and NF-κB expression (Srikantaramas et al. 2007). They possess cytotoxicity on cancer cells mediated through the inhibition of AKT kinase, elevation of the levels of caspase-3 and -9 and Bac/Bcl-2 (Schardl et al. 2004).

**Figure 3.**
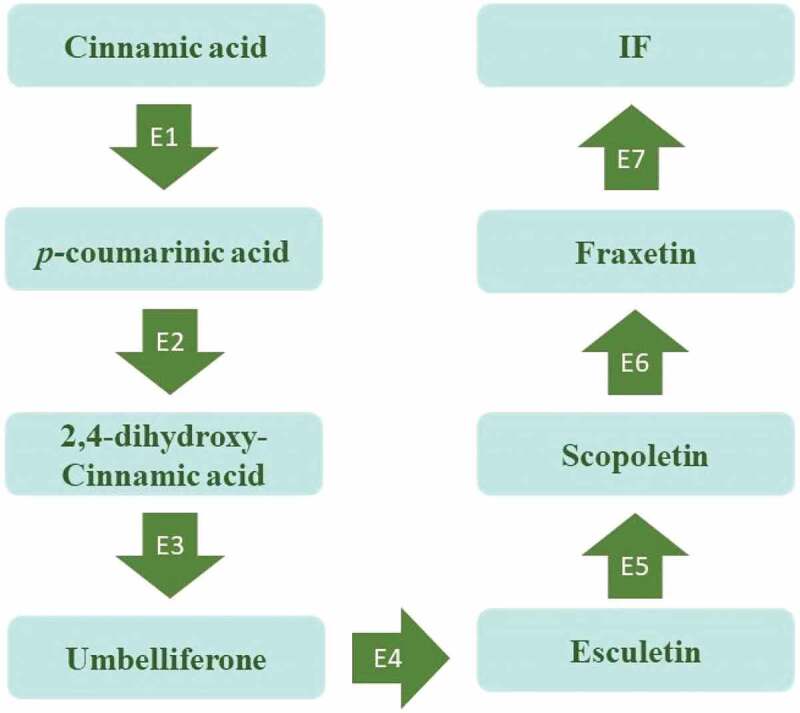
Schematic representation of biosynthesis pharmacological potential of coumarins.

Biosynthesis within the endophytic system occurs via the shikimic acid pathway similar to most of the coumarins. The pathway that yields chorismic acid is a vital branching point for the production of various secondary metabolites. The *ortho*-hydroxylation of cinnamic acid followed by the cis and trans isomerisation further with lactonization of the hydroxyl group leads to the production of the core coumarin structure. This core ring structure further gets hydroxylated (by the action of cinnamate-2-hydroxylase, 4-hydroxylase, 2-oxoglutarate-dependent dioxygenase) producing compounds such as scopoletin, umbelliferone, and isofraxidin or methoxylated by the action of methoxy transferases. However, studies by Brader et al. (2014) demonstrated that isofraxidin acts as a precursor for the biosynthesis of 7-ortho-phenyl ether, which further leads to the synthesis of umbelliferone, aesculetin, scopoletin, and fraxetin. The overall flow of this biosynthesis is given in Figure 3.

### Bergaptenand marmesin

**Figure 4.**
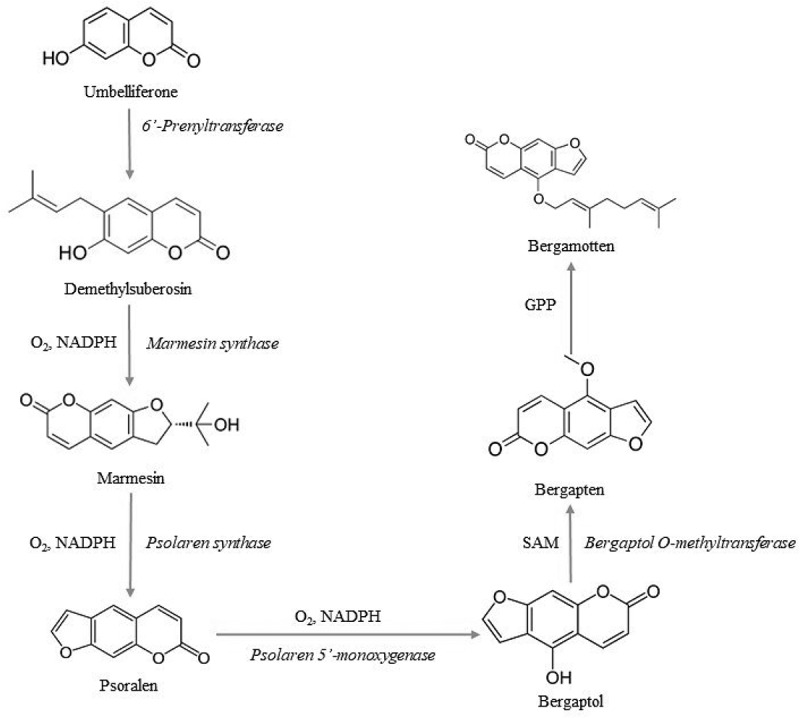
The flow chat of reactions for the production of secondary metabolites.

Bergaptenis produced by the endophytic fungi *Alternaria brassicae, Penicillium* sp., and *Botryodiplodia theobromae* and marmesin is produced by *Fusarium* sp. (Stierle et al. 1993; Debbab et al. 2013). Furocoumarin bergapten is recognised for its potential photosensitising property in the phototherapy of psoriasis and marmesin is known traditionally for its purgative, antisylhillitic, and antihelminthic properties (Stierle et al. 1993; Schardl et al. 2004). They are the tricyclic aromatic compounds from the coumarin class. Most of the naturally occurring forms of bergapten are from the allopsoralen, angelicin, and furo subclasses. In the shikimic acid pathway, after the formation of umbelliferone bergapten production deviates through the mevalonate pathway. The prenylation of this phenylpropanoid at the 6th position produces linear psoralens or 8th position produces angular angelicins. The flow of reactions for the production of both the secondary metabolites is given in Figure 4.

### Scopoletin and umbelliferone

**Figure 5.**
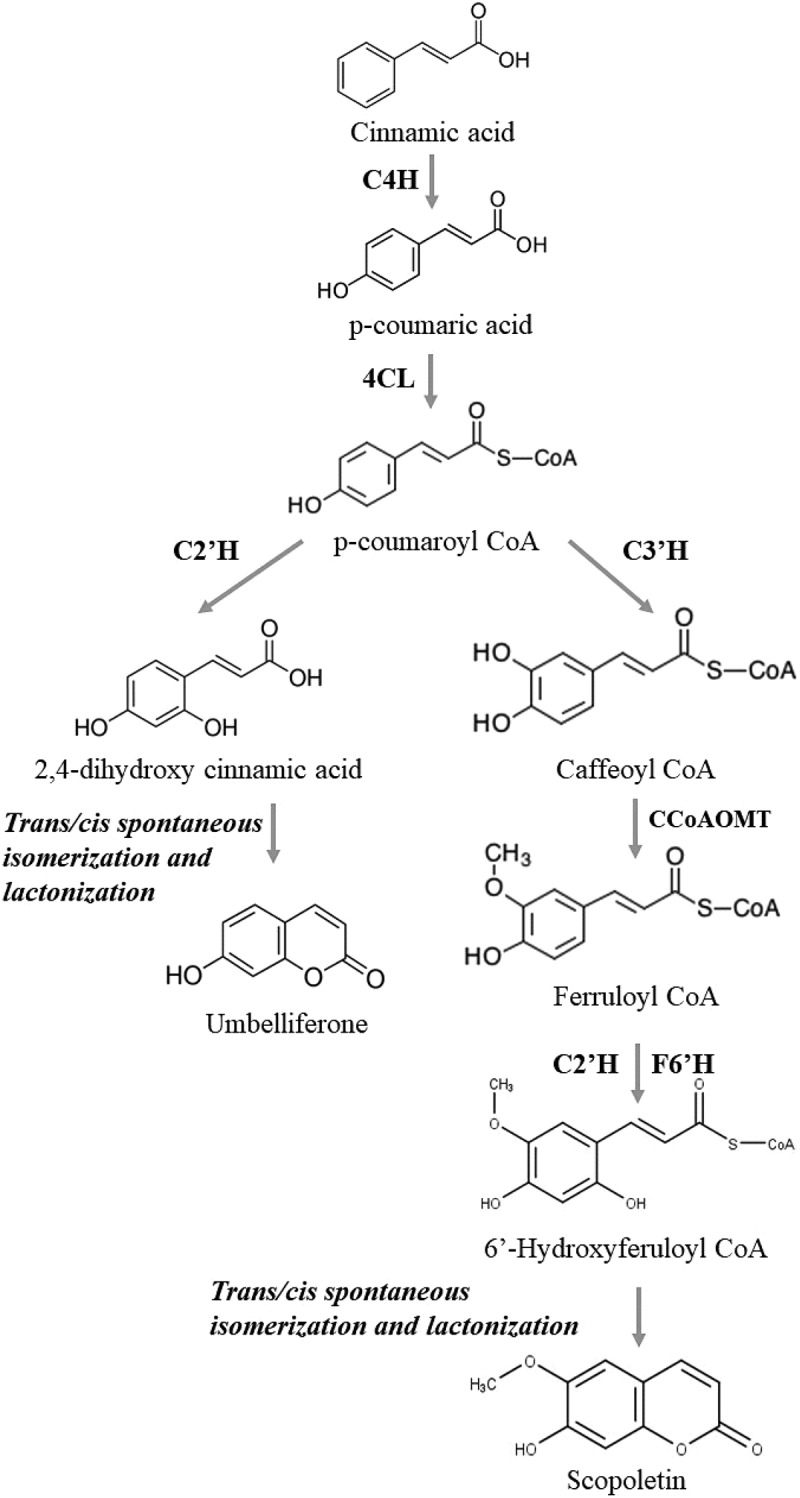
Biosynthesis of scopoletin and umbelliferone.

They are the class of hydroxycoumarins with antitumor and anticancer properties. Scopoletin (6-methoxy-7-hydroxycoumarin) possesses antifungal and anticancer properties by inhibiting acid phosphatase activity in turn resulting in apoptosis. Similarly, Umbelliferone exerts its action as antimicrobial agent and is widely used as a fluorescing compound as an additive in sunscreens (Stierle et al. 1993; Debbab et al. 2013). They are isolated from *Penicillium* sp., *Aegle marmelos*, and *Annulohypoxylon ilanense* (Debbab et al. 2013; Brader et al. 2014; Nicoletti and Fiorentino 2015). The biosynthesis follows the pathway given below (Figure 5).

### Mellein

**Figure 6.**
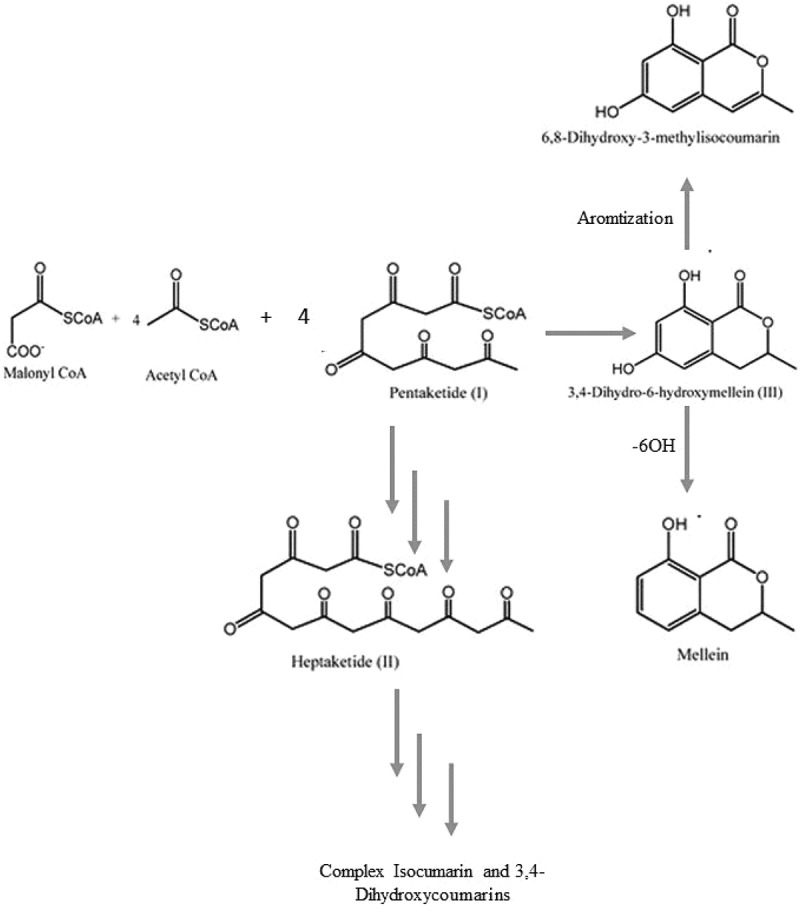
Biosynthesis mechanism of complex isocoumarines or 3,4-dihydroisocoumarins.

Melleins are a class of 3,4-dihydroisocoumarins first reported from *Hydrangea hortensia*, a flowering plant. Melleins and its derivatives have then been isolated from several bacteria, fungi, higher plants, sponges, lichens, and insects. The first fungal endophyte producing this class of secondary metabolite is *Aspergillus melleus* in 1933 and was named ocracin. Its structural analogue was then identified from *Sporormia bipartis*. Furthermore, several compounds derived from this class possess important properties as antimicrobial agents. Their biosynthesis initiates from malonyl CoA that is followed by Claisen condensation with 4 acetyl CoA to form a pentaketide. This pentiketide further undergoes (i) Further chain elongation, spawning the heptaketide (II). Post-PKS modification of II may result in a variety of more complex isocoumarines or 3,4-dihydroisocoumarins. (ii) Cyclisation reaction, which produces the typical six-membered lactone ring synthetising 6-hydroxymellein (III). Further modification of III include aromatisation, generating 6,8-dihydroxy-3-methylisocoumarin, or 6-OH dehydration, forming mullein, as shown in Figure 6.

### Recent advances in coumarin research from endophytic sources

Secondary metabolites from plant and endophytic origin have the ability to alter the host microbiomes. They constitute a varied class of compounds including alkaloids, flavonoids, terpenes, phenolics, isoprenes, steroids, and many others (Stierle et al. 1993; Brader et al. 2014). Coumarins are a part of the phenol family that are non-volatile in nature produced in response to iron deficiency in dicotyledonous plants (Stierle et al. 1993). They have important significance in regulating the microbiome composition of the rhizosphere by extending toxicity against specific pathogenic microorganisms in their effort to outlive their microbial counterpart (Srikantaramas et al. 2007). A recent study by Stierle et al. (1993) demonstrated that beta glucosidase gene knock out in a coumarin-devoid *Arabidopsis* revealed a greater abundance in the presence of Proteobacteria with a corresponding decrease in the Firmicutes near the roots of this plant. Evidence for these findings was provided by Stierle et al. (1993) that suggested the role of coumarins in shaping the SynCom composition as observed by the increased abundance of Pseudomonas in coumarin-deficient *Arabidopsis* mutant type in comparison with that of its wild-type variant. These findings suggest the importance of these endophytes and their corresponding production of coumarins for the host plant.

Biological significance of this plant endophyte is enormous. Recent studies have demonstrated the coumarin derivative, pestalustaine B, obtained by an unprecedented 6/6/5/5-fused tetracyclic system was obtained from the endophyte *Pestalotiopsis asusta* dwelling in the host *Sinopodophyllum hexandrum* (Stierle et al. 1993). The study demonstrated its potent anticancer activity on HeLa, HCT116, and A549 cancer cell lines. Similarly, Stierle et al. (1993) demonstrated that *Pestalotiopsis* sp. also produce isocoumarin derivatives pestalotiopisorin B with potential antimicrobial properties against *Enterococcus faecalis*, MRSA, *E. coli, P. aeruginosa*, and *C. albicans*. Chlorinated and brominated derivatives of dihydroisocoumarins were produced by the endophytic fungi *Lachnum palmae* belonging to the host plant *Przewalskia tangutica* (Stierle et al. 1993). These coumarin derivatives also demonstrated moderate anti-inflammatory activity by inhibiting the NO production in LPS-induced RAW 264.7 cells. Similar to this study, Srikantaramas et al. (2007) demonstrated the cytotoxic effects of Penisarin A and B-sesquiterpene coumarins from *Penicillium isariiforme* against human myeloid leukaemia HL-60, human hepatocellular carcinoma SMMC-7721, lung cancer A-549, breast cancer MCF-7, and human colon cancer SW480 cell lines. They also demonstrated notable inhibition of the NO production suggesting their anti-inflammatory role. Peniisocoumarins, a series of derivatives of coumarins, were isolated from *Penicillium commune* QQF-3 belonging to the host *Kandelia candel* (Brader et al. 2014). They were evaluated for inhibition of α-glucosidase, MptpB inhibition, and its cytotoxic effects against human tumour cell lines A549, HepG2, HeLa, MCF-7, and HEK293T. Comprehensive details of studies towards the isolation of bioactive coumarins and their corresponding biological activities are listed in Table 1.

### Biotransformation of coumarin by endophytes

Technological advances have led to the transformation of chemical compounds with complex structures for improvement of their biological potential (Lv et al. 2013). For instance, Costa et al. (2016) demonstrated that growing *Phanerochaete chrysosporium* in different substrates vary in their metabolite production. In this study, coumarin compound was produced in 8-hydroxycoumarin, 5-hydroxycoumarin, 6-hydroxycoumarin, and 7-hydroxycoumarin. These compounds showed remarkable cytotoxic effects against cancer cell lines including leukaemia cell line (HL-60) (Symeonidis et al. 2009; TabishRehman and U Khan 2015). Study carried out by Lv et al. (2013) showed that imperatorin was transformed by the action of *Penicillium janthinellum* that showed anti-osteoporosis potential on MC3T3-E1 cells. The microbial transformation flow included reduction at α,β-unsaturated lactone ring, hydroxylation, methylation, dehydration, and glycosylation. Intermediates such as (6,7-furano-8-(5a-hydroxyl-methyl-prenyloxy) hydrocoumaric acid and 6,7-furano-8-(2a-hydroxy-3a-en-prenyloxy) hydrocoumaric acid) showed growth promoting ability on MC3T3-E1 cells suggesting these molecules as potent anti-osteoporosis drugs.

Furanocoumarins have demonstrated remarkable anticancer, anti-inflammatory, analgesic, and antispasmodic activities (Schardl et al. 2004). In continuation to this study, Stierle et al. (1993) performed biotransformation of isoimperatorin led to the production of 14-hydroxyl-isoimperatorin, 11-carbonyl-14-hydroxyl isoimperatorin, 11-carbonyl-14-hydroxyl-12,13-dihydrogen-iso-imperatorin, 14-hydroxyl-12,13-dihydrogenisoimperatorin, isoimperatorin-14-O-β-D-mannoside, and isoimperatorin-14-O-β-D-glucoside by the action of *Cunninghamella blakesleana*. The results from this study showed that glycosylation and hydroxylation at C-14 presents the molecule with remarkable anti-osteoporosis activity.

Mousa and Raizada (2013) assessed the conversion of 6′,7′-epoxybergamottin when used as a substrate in submerged fermentation of *Botrytis cinerea, Geotrichum citri, Lasiodiplodia theobromae, Penicillium digitatum, Penicillium ulaiense*, and *Phomopsis citri*. It was observed that four derivates of coumarin namely, 6′,7′-dihydroxybergamottin, bergaptol, and an opened lactone ring metabolite 6,7-furano-5-(6′,7′-dihydroxy geranyloxy)-2-hydroxy-hydrocoumaric acid were obtained with *Penicillium ulaiense* being the most potent. Also, they identified that this biotransformation required 6′,7′-epoxybergamottin as an inducing agent in the synthesis on coumarin derivatives. In a similar study, the bio-transforming ability of *Aspergillus niger, A. niger, A. terreus, A. flavus, A. ochraceus*, and *Aspergillus fumigatus* were evaluated in submerged and resting cultures (Kusari et al.). The study demonstrated that resting cells produced 5-hydroxycoumarins as the main product of biotransformation. Within a span of 7 days, dihydrocoumarin, 6-hydroxy-3,4-dihydrochromen-2-one (4) and 2-(3-hydroxypropyl)-phenol were obtained from *A. flavus* and *A. niger* being the most potent. Several such biotransformation are being attempted to improvise the production yield and enhance biological activity of many coumarin derivatives.

### Perspectives and conclusion

Plant-microbial association is known to the mankind since time immemorial. Yet, only recently the association between the host and its endophyte responsible for the production of plant secondary metabolite mimicking the host counterpart has been identified. In that, several secondary metabolites responsible for plant defence are produced by the endophytic microorganism to escape host defence and therefore dwell within the plant as an endophyte without eliciting immune response. Although much research has identified several fungal endophytes producing coumarins and its derivatives as secondary metabolites, very few studies have indicated such phytochemical isolation from bacterial sources. However, the exact reason for such disparity is unknown. In addition, the mechanism employed by the endophyte to produce metabolites mimicking the products of the host system is also not well understood. Several attempts to unravel these pathways have failed to identify specific genes that work in tandem for secondary metabolite production. The present review highlights this host–endophytic interaction and the corresponding production of one of the most commonly known secondary metabolite- coumarin and its derivatives. The derivatives of coumarin and their biological properties are also review in this article with an emphasis on the biotransformation potential of the derivatives. The observations from the review indicate that isolation and industrial production of these coumarins face several challenges in terms of large-scale production. Microbial production system depends on the optimisation of culture conditions; the overall production system is at times problematic because both the host and the endophyte contribute to the synthesis of the metabolite *in planta*, whereas the production system has seldom effects from the host. Chances are that the endophyte culture without its host might not even produce the desired compound or produce it in a very small concentration resulting in inefficiency of its industrial production. To overcome such difficulties, transgenic technology can be employed that results in a sustainable and cost-effective strain of the endophyte with better efficiency. In addition, a better understanding of the biosynthetic pathway that is the cross-talk between the plant and its endophyte that may help understand the regulation mechanism. With this knowledge, a production system can be established with a regulated production of the desired metabolite. Using the host plant with the endophyte for the large-scale production system is another least exploited area of research, which can be taken up to assess the efficiency of a combined production system. Overall, a careful understanding of the mechanism of coumarin biosynthesis with intricate details regarding the host–endophyte cross-talk will provide good information on the industrial optimisations for large-scale production of this secondary metabolite.
